# Automatic Estimation of Interpersonal Engagement During Naturalistic Conversation Using Dyadic Physiological Measurements

**DOI:** 10.3389/fnins.2021.757381

**Published:** 2021-10-26

**Authors:** Iman Chatterjee, Maja Goršič, Joshua D. Clapp, Domen Novak

**Affiliations:** ^1^Department of Electrical Engineering and Computer Science, University of Cincinnati, Cincinnati, OH, United States; ^2^Department of Psychology, University of Wyoming, Laramie, WY, United States

**Keywords:** affective computing, conversation, dyads, hyperscanning, interpersonal interaction, physiological computing, physiological synchronization, psychophysiology

## Abstract

Physiological responses of two interacting individuals contain a wealth of information about the dyad: for example, the degree of engagement or trust. However, nearly all studies on dyadic physiological responses have targeted group-level analysis: e.g., correlating physiology and engagement in a large sample. Conversely, this paper presents a study where physiological measurements are combined with machine learning algorithms to dynamically estimate the engagement of individual dyads. Sixteen dyads completed 15-min naturalistic conversations and self-reported their engagement on a visual analog scale every 60 s. Four physiological signals (electrocardiography, skin conductance, respiration, skin temperature) were recorded, and both individual physiological features (e.g., each participant’s heart rate) and synchrony features (indicating degree of physiological similarity between two participants) were extracted. Multiple regression algorithms were used to estimate self-reported engagement based on physiological features using either leave-interval-out crossvalidation (training on 14 60-s intervals from a dyad and testing on the 15th interval from the same dyad) or leave-dyad-out crossvalidation (training on 15 dyads and testing on the 16th). In leave-interval-out crossvalidation, the regression algorithms achieved accuracy similar to a ‘baseline’ estimator that simply took the median engagement of the other 14 intervals. In leave-dyad-out crossvalidation, machine learning achieved a slightly higher accuracy than the baseline estimator and higher accuracy than an independent human observer. Secondary analyses showed that removing synchrony features and personality characteristics from the input dataset negatively impacted estimation accuracy and that engagement estimation error was correlated with personality traits. Results demonstrate the feasibility of dynamically estimating interpersonal engagement during naturalistic conversation using physiological measurements, which has potential applications in both conversation monitoring and conversation enhancement. However, as many of our estimation errors are difficult to contextualize, further work is needed to determine acceptable estimation accuracies.

## Introduction

Effective interpersonal communication is essential to many aspects of social functioning and human growth. For example, teacher-student engagement is critical to instruction (Lee, 2012; Quin, 2017), therapist-client alliance is vital for mental health intervention (Sharf et al., 2010; Flückiger et al., 2012), and a clear understanding of the needs and desires of others is vital for effective conflict resolution (Overall and McNulty, 2017). By contrast, the disruption of communication due to, e.g., misunderstanding or lack of trust, can contribute to a range of negative outcomes. However, as even trained professionals sometimes have trouble recognizing the moods, needs and desires of their conversation partner, there is a great need for technologies that could automatically quantify the level of interpersonal engagement in pairs or groups. Such technologies could be used as a complement to self-report measures and external observation when analyzing communication scenarios and could potentially be used for real-time feedback: providing communication participants with information about others’ engagement levels, allowing them to intelligently adapt their own behavior to improve engagement and overall communication outcome (Schilbach, 2019; Järvelä et al., 2020; Pan and Cheng, 2020).

Interpersonal engagement (i.e., the degree to which both participants are interested in and actively participating in a conversation) could be automatically quantified through analysis of physiological data of both participants – for example, heart rate and respiration. In the areas of affective computing and psychophysiology, physiological data of individuals have already been used to identify diverse mental states: for example, stress and distraction in drivers and pilots (Healey and Picard, 2005; Haarmann et al., 2009), boredom and frustration in computer game players (Liu et al., 2009; Chanel et al., 2011) or engagement in patients undergoing rehabilitation (Rodriguez-Guerrero et al., 2017). To identify these states, physiological responses are combined with pattern recognition algorithms (mostly based on supervised machine learning) that take multiple physiological features (e.g., mean heart rate, heart rate variability) as inputs, then output either a discrete psychological class (e.g., frustrated/bored/engaged) or a value on a continuous scale (e.g., boredom of 63 on a 0-100 scale) (Novak et al., 2012; Aranha et al., 2019). In dyadic and group settings, a similar approach could be used to quantify interpersonal engagement based on physiological data from more than one participant.

In such dyadic and group situations, automatic quantification of engagement would not need to be only based on individuals’ physiological responses. It could also leverage the concept of physiological synchrony: a phenomenon in which the physiological responses of two or more individuals gradually converge as they interact. Synchrony occurs involuntarily as a function of interpersonal dynamics (Pérez et al., 2017; Haataja et al., 2018), and larger group-level studies have found that the degree of synchrony is correlated with, e.g., perceived therapist empathy and alliance in therapist–client interactions (Finset and Ørnes, 2017; Bar-Kalifa et al., 2019; Kleinbub et al., 2019; Tschacher and Meier, 2020) and overall engagement in teachers and students (Dikker et al., 2017; Bevilacqua et al., 2018; Sun et al., 2020; Zheng et al., 2020). As physiological synchrony can be quantified using metrics such as correlation and cross-mutual information (Helm et al., 2018; Schneider et al., 2020), it could easily be combined with individual physiological features in a pattern recognition algorithm, potentially providing additional information about the dyad.

However, while there have been many studies targeting group-level analysis of physiological synchrony (e.g., correlating synchrony and engagement in a large sample), there has been relatively little work on quantifying engagement or other interpersonal states at the level of individual dyads (e.g., tracking interpersonal engagement of a specific dyad over time). A handful of studies have used classification algorithms with a single physiological modality (e.g., electroencephalography alone) to discriminate between two states (e.g., engaged vs. unengaged dyads) (Hernandez et al., 2014; Konvalinka et al., 2014; Muszynski et al., 2018; Zhu et al., 2018; Brouwer et al., 2019; Pan et al., 2020) with one study discriminating between four affective states (Verdiere et al., 2019). A final study used regression algorithms to map physiological synchrony to self-reported arousal and valence on 1–9 scales using electroencephalography during video watching (Ding et al., 2021). To our knowledge, only one study has attempted to combine multiple physiological signals to quantify interpersonal engagement: our own previous work, done in a competitive gaming context (Darzi and Novak, 2021).

In the current study, we measured multiple physiological signals, extracted both individual and synchrony features, and used this information together with multiple regression algorithms to quantify the degree of self-reported interpersonal engagement during continuous conversation. For purposes of this study, engagement was defined as the degree to which participants are actively participating in and interested in the conversation, similarly to definitions of engagement in, e.g., teacher–student dyads (Carroll et al., 2020) or human interaction with technology (Zimmerli et al., 2013). The study goes beyond the state of the art by performing dyad-level automated engagement estimation rather than large group-level analyses, potentially providing a method to dynamically estimate dyadic engagement in conversation settings such as mental health counseling. Furthermore, it goes beyond the state of the art by combining information from multiple physiological signal modalities rather than focusing on a single modality.

## Materials and Methods

### Study Protocol and Self-Report Measures

The study was approved by the University of Wyoming Institutional Review Board. Data collection took place between October and December 2020, with participants recruited among students and staff of the University of Wyoming. Due to the COVID-19 pandemic, participants were encouraged to volunteer for the study in self-selected dyads (e.g., friends); however, if individual participants volunteered for the study, they were paired with another available individual participant. No rules were placed on valid pairs.

Each dyad took part in a single 1-h session. Upon arrival, the purpose and procedure of the experiment were explained, and participants provided informed consent. They then provided demographic information and completed self-report measures on four traits known to influence physiological synchrony (McKillop and Connell, 2018; Steiger et al., 2019; Sachs et al., 2020): cognitive and affective empathy with the Questionnaire of Cognitive and Affective Empathy (QCAE) (Reniers et al., 2011), social anxiety with the Brief Fear of Negative Evaluation Scale (BFNES) (Leary, 1983), and depression with the Center for Epidemiologic Studies Depression Scale (CESD) (Radloff, 1977).

**FIGURE 1:**
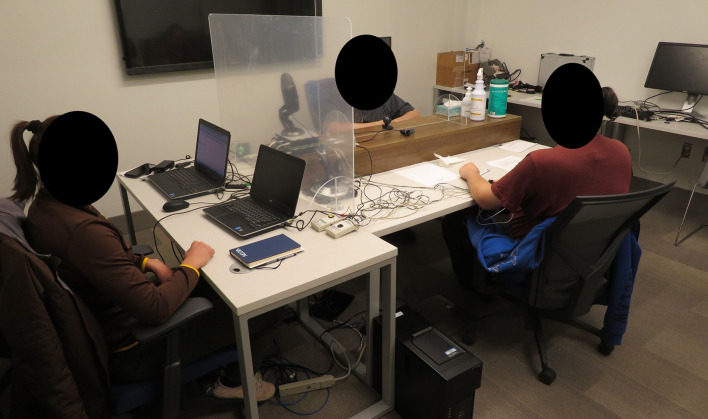
Two participants in the study protocol, sitting facing each other separated by a plexiglass barrier. The experimenter sits to the side with the data collection laptops.

Participants were then seated approximately 1.5 m apart, facing each other, separated by a transparent plexiglass barrier, with the experimenter sitting to their side (Figure 1). They removed their face masks and self-applied physiological sensors (see next section) while visually supervised by the experimenter, who provided instructions and feedback. Physiological signal quality was visually checked, and corrections were made as necessary until good quality was obtained. We acknowledge that the self-application process likely introduced greater signal variability than the standard approach of having the experimenter apply sensors, particularly in the electrocardiogram (which has more possible ways to place the electrodes). However, such self-application was a requirement by the Institutional Review Board to maintain social distancing during the COVID-19 pandemic.

Following sensor attachment, participants rested quietly for 2 min with eyes closed to obtain baseline physiological measurements. They then engaged in approximately 15 min of conversation; they were instructed by the experimenter to begin by discussing each other’s career goals and aspirations, but were allowed to switch topics as desired. After 60 s, the experimenter silently raised their hand, which served as a visual cue for participants to mark their engagement level over the previous 60 s on a visual analog scale ranging from “none” to “very high.” Ratings for each 60-s period were recorded on a separate piece of paper that participants then set aside to avoid potential influences on subsequent ratings. Participants were instructed to self-report engagement as “the degree to which they were interested in and actively participating in the conversation.” They were asked in advance to self-report engagement without breaking up conversation if possible; for example, by looking down and making a mark quickly while continuing to talk or listen. The experimenter visually watched to see when participants had finished reporting engagement, and then restarted a 60-s timer to indicate the start of a new interval. The conversation continued until 15 60-s intervals had been completed. Ratings on all visual analog scales were converted to numerical engagement scores (0–100) for analysis.

At the end of the study, participants filled out the Self-Assessment Manikin (SAM) (Bradley and Lang, 1994), which measures individual valence, arousal and dominance, as well as the Interpersonal Interaction Questionnaire (IIQ) (Goršič et al., 2019), which assesses the amount, balance and valence of dyadic conversation. Both questionnaires were completed with respect to the overall 15-min conversation. Participants then removed the physiological sensors and were reimbursed $15 for their involvement in the study.

Throughout the study protocol, audio and video of both participants were collected using a Yeti X microphone (Blue Microphones, United States) and two consumer-grade webcams. After the session, a member of the research team (co-author Goršič) watched the videos and rated dyad engagement for each 60-s interval. This coder did not have access to participants’ self-report ratings or physiological data prior to assigning codes.

### Physiological Sensors

Two g.USBamp biosignal amplifiers (g.tec Medical Engineering GmbH, Austria) and associated sensors were used to collect four physiological signals from each participant. The electrocardiogram (ECG) was measured using four disposable electrodes placed on the trunk in a configuration recommended by g.tec: one electrode on the left part of the chest, one on the right part of the chest, one on the left part of the abdomen, and a ground electrode on the upper left part of the back. Skin conductance was measured using the g.GSRsensor2 sensor, which includes two dry electrodes placed on the distal phalanges of the index and middle fingers of the non-dominant hand. Respiration was measured using a thermistor-based respiration airflow sensor placed below the nose and in front of the mouth. This sensor is essentially a thin white wire and was chosen to minimize occlusion of the face and thus effect on dyad engagement. Finally, peripheral skin temperature was measured using the g.Temp sensor, which includes a single dry electrode placed on the distal finger of the non-dominant hand.

All signals were sampled at 600 Hz, and an analog 60-Hz notch filter was applied to them. The ECG was additionally filtered with an analog 0.1-Hz highpass filter while the other three signals were additionally filtered with an analog 30-Hz lowpass filter. The amplifiers were synchronized to each other via a synchronization cable and MATLAB/Simulink model provided by g.tec. Video from the cameras and microphones was synchronized to physiological amplifiers via simultaneous manual button press in both video and physiology interfaces.

### Physiological Feature Extraction

Each dyad’s physiological signals were segmented into individual intervals: the 2-min baseline interval and 15 60-s conversation intervals. The brief engagement self-reporting periods between the 60-s intervals were not included in analysis. The skin conductance, respiration, and skin temperature signals were filtered with fourth-order Butterworth lowpass filters with cutoff frequencies of 5 Hz. Peak detection algorithms were used to identify peaks in the ECG corresponding to individual R-waves (heartbeats) as well as peaks in the respiration signal corresponding to individual breaths. All detected peaks in the ECG were visually inspected, and both false positives and false negatives were manually corrected as needed. If the researcher was not able to identify the precise location of an R-wave due to noise, one was interpolated halfway between two neighboring two valid R-waves. This occurred in approximately 1–2% of R-waves. Finally, a peak detection algorithm was used to identify individual skin conductance responses (SCRs) in the skin conductance signal. SCRs were defined as brief transient increases in skin conductance whose peak occurs less than 5 s after the beginning of the increase and whose amplitude (from beginning to peak) is at least 0.05 microsiemens (Boucsein, 2012).

After filtering and peak detection, multiple features were extracted from each 60-s conversation interval and from the baseline interval. These features can be divided into individual physiological features (calculated from a single participant’s physiological signal) and synchrony features (calculated from both participants’ corresponding signals – e.g., respiration of both participants).

The individual physiological features on each interval were:

– ECG: The mean heart rate and three time-domain metrics of heart rate variability (the standard deviation of interbeat intervals, the root-mean-square (RMS) value of successive interbeat interval differences, and the percentage of successive interbeat intervals that differ by more than 50 ms). These metrics are standard and well-defined in the literature (Task Force of the European Society of Cardiology and the North American Society of Pacing and Electrophysiology, 1996).
– Skin conductance: The mean skin conductance level, the difference between the initial and final skin conductance, the number of SCRs, and the mean SCR amplitude.
– Respiration: the mean respiration rate and the standard deviation of respiratory periods.
– Peripheral skin temperature: the mean skin temperature and the difference between the initial and final skin temperature.

The synchrony features were calculated from instantaneous heart rate and instantaneous respiration rate signals (i.e., heart/respiration rate as a function of time, calculated from raw ECG and respiration using the same procedure as in our previous work; Darzi and Novak, 2021) as well as from raw skin conductance and skin temperature signals. They were:

– Dynamic time warping distance, using the same procedure as Muszynski et al. (2018). This approach uses dynamic programming to quantify the similarity between two signals, and allows some temporal flexibility with regard to, e.g., temporal delays between events in individual participants’ signals (Hernandez et al., 2014).
– Non-linear interdependence, using the same procedure as Muszynski et al. (2018). This feature measures the geometrical similarity between the state space trajectories of two dynamical systems, and involves applying time-delay embedding to the two measured signals to reconstruct trajectories analogous to shape distribution distance (Muszynski et al., 2018).
– Coherence, using the same procedure as our previous work (Darzi and Novak, 2021). Coherence is a standard signal processing method that finds the co-oscillation of two signals in one or multiple frequency bands, and was calculated in different frequency ranges for different signals. For example, respiration coherence was calculated in the 0–2 Hz band while heart rate coherence was calculated in 0.1–0.15 Hz and 0.15–0.4 Hz bands (Darzi and Novak, 2021).
– Cross-correlation, using the same procedure as our previous work (Darzi and Novak, 2021). This is essentially a Pearson correlation between the two participants’ signals, and is thus a very simple measure that is vulnerable to, e.g., temporal delays and non-linearity (Hernandez et al., 2014; Schneider et al., 2020).

Instantaneous heart rate and respiration rate signals were used since they are expected to better synchronize between participants than raw ECG and respiration signals (Darzi and Novak, 2021). However, no such extracted signals are defined in the literature for skin conductance or skin temperature, and raw signals were used in those cases.

### Estimation of Interpersonal Engagement

In this section, we first describe the overall framework of engagement estimation (see section “Overall Problem Framework”), followed by a description of two primary analyses: dyad-specific (see section “Dyad-Specific Engagement Estimation”) and dyad-non-specific (see section “Dyad-Non-Specific Engagement Estimation”) engagement estimation. Additionally, we describe two secondary analyses to determine whether removing some data types decreases estimation accuracy (see section “Secondary Analysis: Engagement Estimation Without Synchrony or Participant Characteristics”) and whether personality has an effect on estimation accuracy (see section “Secondary Analysis: Effect of Personality on Engagement Estimation”).

#### Overall Problem Framework

The collected data consist of multiple physiological features (see previous section), multiple participant characteristics (age, gender, personality traits), and each participant’s self-reported engagement on a scale of 0–100. There are 16 data points per dyad: the baseline and 15 60-s conversation intervals. The overall goal of automated engagement estimation is to determine engagement in a particular interval based on physiological features from that interval.

As each conversation interval includes two self-reported engagement values from the two individual participants, we first calculated dyad engagement in each interval as the mean of the two individual values. This dyad engagement was then used as the variable to be estimated. The underlying assumption is that both participants are rating the same phenomenon (conversation engagement) and are able to rate it accurately. We acknowledge that this is not necessarily suitable for situations where the two participants have different impressions of current engagement, and discuss this further in the section “Discussion.”

Since dyad engagement is on a scale of 0–100, the engagement estimation represents a regression problem rather than classification problem (Novak et al., 2012; Aranha et al., 2019), and can be solved with many possible regression algorithms. Whatever the algorithm, its ‘error’ for an individual interval would be determined as the difference between the engagement value estimated by the algorithm and the ‘reference’ value self-reported by the participants. The performance of the algorithm over multiple conversation intervals can be determined by averaging the error over those intervals. For purposes of this study, we used two averaging methods: the mean absolute (MA) difference between estimated and reference engagement values (MA error) and the RMS value of the difference between estimated and reference engagement values (RMS error). Both are standard error metrics in regression problems, with RMS giving a relatively higher weight to large individual errors. However, both RMS and MA errors should be contextualized with regard to other approaches: e.g., in our case, non-physiological methods of engagement estimation.

In affective computing, regression algorithms are commonly trained using previously recorded and labeled data – i.e., supervised machine learning (Novak et al., 2012; Aranha et al., 2019). Our study used three supervised machine learning methods as engagement estimators: a binary decision tree, least-squares boosting, and random forest. All were implemented using standard functions in MATLAB 2020b (Mathworks, United States): *fitrtree*, *fitensemble*, and *treebagger*. They were chosen due to their ability to handle non-linear problems, as the relationship between physiological features and engagement was expected to be strongly non-linear. For full disclosure: two additional machine learning methods (multilinear perception and stepwise linear regression) were also evaluated, but both achieved systematically worse results than the three above methods and are thus not discussed further.

In affective computing, algorithms for psychological state estimation are commonly either trained using existing data from the same individual (“person-specific”) or using data from other individuals (“person-non-specific” or “person-independent”) (Novak et al., 2012; Aranha et al., 2019). Both approaches have advantages and disadvantages: training using data from the same individual may allow more personalized estimation, but may not be practical in situations where each person only interacts with a machine once or sporadically. We thus conducted two primary analyses focusing on dyad-specific estimation and dyad-non-specific estimation.

#### Dyad-Specific Engagement Estimation

The analysis examined whether the engagement of a dyad can be estimated given training data from the same dyad. Thus, engagement estimation algorithms were trained for each dyad separately using the principle of leave-interval-out crossvalidation: they were trained on 14 conversation intervals, then tested on the remaining interval. This was repeated 15 times, with each conversation interval serving as the ‘test’ interval once, and RMS and MA errors were then calculated over the 15 test intervals. This approach is commonly used in single-user affective computing when multiple measurements are available from each participant and there are a limited number of participants or significant variability between participants (Novak et al., 2012; Aranha et al., 2019).

In addition to the machine learning methods, a ‘baseline’ method was also used: to obtain engagement for the test interval, simply take the median value of reference engagement in the other 14 intervals. This does not take physiological data into account and allows us to contextualize the accuracy of the machine learning methods with respect to a basic method.

#### Dyad-Non-Specific Engagement Estimation

In the second primary analysis, we used the principle of leave-dyad-out crossvalidation: engagement estimation algorithms were trained on all data from all but one dyad and then tested on all 15 intervals of the remaining dyad. The procedure was repeated as many times as there were dyads, with each dyad serving as the ‘test’ dyad once, and RMS and MA errors were then calculated over all test dyads. All physiological features from the 15 intervals were ‘normalized’ by subtracting the value of that feature from the baseline period; this is a common approach to reduce intersubject variability in single-user affective computing (Novak et al., 2012; Aranha et al., 2019). Leave-dyad-out crossvalidation is expected to yield a lower accuracy than dyad-specific estimation due to larger variability and lack of training data from the analyzed dyad (Novak et al., 2012; Aranha et al., 2019).

In addition to physiological features, dyad-non-specific algorithms also included each participant’s age, gender (coded as 0 = male, 1 = female, 2 = non-binary), and four personality traits (social anxiety, depression, cognitive empathy, affective empathy) as additional inputs. Our hope was that they may help the regression algorithms better compensate for inter-dyad differences, as seen in our previous single-user work (Darzi et al., 2019); they were not included in dyad-specific estimation since they are the same for all 15 intervals of the same dyad.

A ‘baseline’ method was again used: to obtain engagement for all 15 intervals of a dyad, simply take the median value of reference engagement across all other dyads. Furthermore, as a second basis for comparison, we evaluated the ability of the external coder to accurately estimate engagement. This was done by calculating the same MA and RMS errors between reference (self-reported) engagement and the engagement ratings provided by the external coder based on audio and video recordings. This was considered a reasonable comparison to the dyad-non-specific rather than dyad-specific estimation since the external coder would also not have access to engagement ratings from the current dyad.

Finally, we used predictor importance algorithms in the three MATLAB functions to identify the most important features for engagement estimation using the three machine learning methods. As dyad-non-specific estimation involved as many models as there were dyads, the most important features were identified for each individual model and then averaged across the models to obtain the overall most important features. This was done only for dyad-non-specific rather than dyad-specific estimation since the large number of models (number of dyads × 15 intervals) was expected to result in too much variability in predictor importance.

#### Secondary Analysis: Engagement Estimation Without Synchrony or Participant Characteristics

In both primary analyses, we used all available data to estimate engagement. However, physiological synchrony features require more computation to obtain compared to individual physiological features, and personality traits must be collected using potentially long self-report measures. Thus, they should only be included if they improve the estimation accuracy.

In this secondary analysis, we first repeated dyad-specific engagement estimation (see section “Dyad-Specific Engagement Estimation”) with physiological synchrony removed from the input dataset. We then repeated the dyad-non-specific engagement estimation (see section “Dyad-Non-Specific Engagement Estimation”) with physiological synchrony (but not participant characteristics) removed from the input dataset, and finally with participant characteristics (but not synchrony) removed from the input dataset. Each of these is expected to lead to lower accuracy due to fewer available features. If no decrease in accuracy is observed, this would indicate that the removed features do not contain additional information or that the number of features is too high for the machine learning algorithms to handle, leading to overfitting.

#### Secondary Analysis: Effect of Personality on Engagement Estimation

Finally, since the measured personality traits (cognitive empathy, affective empathy, social anxiety, depression) are known to influence physiological synchrony (McKillop and Connell, 2018; Steiger et al., 2019; Sachs et al., 2020), they may also influence the degree to which engagement can be estimated from physiological measurements. For each dyad, we thus calculated the mean value of each trait among both participants in the dyad and the difference between the values of each trait among both participants in the dyad. Spearman correlations were then calculated between these trait values and the RMS and MA errors obtained with the most accurate machine learning method in both primary analyses.

## Results

### Participants

Eighteen dyads volunteered and all completed the study protocol. Upon manual inspection of self-reported engagement, two dyads were found to generally disagree on engagement ratings and were thus removed – since reference dyad engagement is the mean of the engagement values reported by the individuals in the dyad, it was considered unreliable for these two dyads. This left 16 valid dyads. For these 16 dyads, all physiological features, self-reported engagement, and personality data were available.

Of the 16 dyads, 13 self-described as friends, 1 as being in a relationship, and 2 as strangers. There were two female–female dyads, nine male–male dyads, four male–female dyads, and one dyad where both participants identified as non-binary. Their age was 20.4 ± 2.5 years (mean ± standard deviation), with the range being 18–28 years. Their personality scores were: 55.5 ± 9.6 for cognitive empathy (possible range 19–95), 32.3 ± 4.7 for affective empathy (possible range 12–60), 36.9 ± 8.2 for social anxiety (possible range 12–60), and 17.0 ± 10.6 for depression (possible range 0–60). In all cases, higher scores indicate higher empathy/anxiety/depression.

We first characterize dyads’ conversations by presenting engagement values and IIQ and SAM results in section “Summary of Conversations.” Results of the two primary analyses are presented in sections “Dyad-Specific Engagement Estimation” and “Dyad-Non-Specific Engagement Estimation,” followed by results of secondary analyses in sections “Secondary analysis: Engagement Estimation Without Synchrony or Participant Characteristics” and “Secondary analysis: Effect of Personality on Engagement Estimation.” As most results did not follow a normal distribution, they are presented in the form of median (25th percentile – 75th percentile).

### Summary of Conversations

Self-reported engagement values across all dyads and intervals were 76 (64–90). Within each dyad, the engagement range (difference between maximum and minimum value reported by the dyad) was 31 (22–39). As mentioned, each self-reported engagement value for an interval (used in further analysis) is the mean of the two values given by the two individuals in the dyad for that interval. The absolute difference in self-reported engagement for a given interval between the two individuals in the dyad, across all dyads and intervals, was 13.5 (7–23). The intraclass correlation (ICC) for concordance of engagement ratings made by dyad members across all assessment periods was also examined. ICCs provide a more stringent test of consistency than standard Pearson correlations in that estimates account for both the covariation and absolute agreement of continuous scores. A one-way, random-effects model for ratings in these data returned an ICC = 0.46, consistent with acceptable levels of agreement within dyads (Cicchetti, 1994).

On the SAM, participants rated their valence over the 15-min period as 3 (2–3), arousal as 4 (2.75–4.25), and dominance as 3 (2–4); all three have a range of 1–9, with 1 indicating highest valence/arousal/dominance. On the IIQ, participants rated the overall amount of conversation over the 15-min period (mean of questions 1 and 2 on IIQ) as 4 (3.5–4.25) on a 1–5 scale, with 5 indicating constant conversation. Participants rated the overall conversation valence as 5 (4–5) on a 1–5 scale, with 5 indicating very high valence.

### Dyad-Specific Engagement Estimation

Table 1 shows RMS and MA errors for the different machine learning methods and the ‘baseline’ estimator (median engagement of other 14 intervals).

**TABLE 1:** Medians and interquartile ranges of root-mean-square (RMS) and mean absolute (MA) errors for the baseline estimator (median of other 14 intervals) and for three machine learning methods in dyad-specific engagement estimation.

	**Baseline (median)**	**Binary decision tree**	**Least squares boosting**	**Random forest**
RMS error	8.1 (6.3–11.2)	8.1 (6.4–10.3)	8.1 (6.5–10.6)	8.5 (6.6–10.3)
MA error	6.6 (4.9–7.9)	6.5 (5.2–8.0)	6.4 (5.0–8.2)	6.3 (5.3–8.2)

### Dyad-Non-Specific Engagement Estimation

Table 2 shows RMS and MA errors for the different machine learning methods and the ‘baseline’ estimator (median engagement of other 15 dyads). The top five most important features for each of the three machine learning methods are listed in Table 3.

**TABLE 2:** Medians and interquartile ranges of root-mean-square (RMS) and mean absolute (MA) errors for the baseline estimator (median of other 17 dyads) and for three machine learning methods in dyad-non-specific engagement estimation.

	**Baseline (median)**	**Binary decision tree**	**Least squares boosting**	**Random forest**
RMS error	14.0 (13.8–14.2)	13.1 (10.5–18.4)	12.3 (11.3–15.8)	12.6 (10.1–14.7)
MA error	11.6 (11.5–11.7)	10.0 (8.8–13.2)	10.2 (8.8–13.5)	10.7 (8.4–13.2)

**TABLE 3:** The top five most important features for dyad-non-specific engagement estimation using each of the three machine learning methods.

	**Binary decision tree**	**Least squares boosting**	**Random forest**
1	Mean heart rate of P1	Mean heart rate of P1	Mean heart rate of P1
2	Respiration coherence	Respiration coherence	Heart rate cross-correlation
3	Heart rate cross-correlation	RMSSD of P2	Respiration coherence
4	pNN50 of P1	Respiration discrete time warping distance	pNN50 of P1
5	SD of respiratory periods of P1	RMSSD of P1	Mean respiration rate of P2

*P1, participant 1; P2, participant 2; RMSSD, root-mean-square of successive interbeat interval differences; pNN50, percentage of successive interbeat intervals that differ by more than 50 ms, SD, standard deviation.*

In this analysis, we also planned to compare how accurately self-reported engagement could be estimated by the external coder based on audio and video recordings. Due to a technical issue, recordings from 4 dyads were lost, and this part of the analysis was only done with 12 dyads. The external coder achieved an RMS error of 15.2 (11.5–17.3) and MA error of 13.6 (9.7–15.7). For comparison, when calculating errors only over these 13 dyads, the least-squares boosting algorithm achieved an RMS error of 11.8 (10.9–15.1) and MA error of 9.7 (8.7–12.6) while the baseline (median-based) estimator achieved an RMS error of 14.0 (13.8–14.2) and MA error of 11.6 (11.4–11.8).

### Secondary Analysis: Engagement Estimation Without Synchrony or Participant Characteristics

If physiological synchrony features are removed from the input dataset, the most accurate algorithm in dyad-specific estimation is the binary decision tree, with an RMS error of 8.1 (6.4–11.1) and MA error of 6.5 (5.0–8.6). The most accurate algorithm in dyad-non-specific estimation is the random forest, with an RMS error of 13.7 (11.0–18.6) and MA error of 12.0 (9.7–16.3).

If participant characteristics are removed from the input dataset, the most accurate algorithm in dyad-non-specific estimation is the random forest, with an RMS error of 14.0 (12.3–15.3) and MA error of 12.4 (10.6–15.3).

### Secondary Analysis: Effect of Personality on Engagement Estimation

As the least-squares boosting exhibited the lowest errors in both dyad-specific and dyad-non-specific estimation (Tables 1, 2), RMS and MA errors obtained with this method were used to calculate Spearman correlations with regard to both dyad-specific and dyad-non-specific estimation.

Table 4 shows correlations between personality traits and RMS and MA errors obtained in dyad-specific estimation while Table 5 shows correlations between personality traits and RMS and MA errors obtained in dyad-non-specific estimation. In dyad-specific estimation, significant correlations can be seen between RMS error and affective empathy and depression as well as between MA error and affective empathy. In dyad-non-specific estimation, a significant correlation can be seen between RMS error and depression.

**TABLE 4:** Correlations between engagement estimation errors and the means and differences in four personality traits in dyad-specific estimation.

	**SA mean**	**SA diff**	**AE mean**	**AE diff**	**CE mean**	**CE diff**	**D mean**	**D diff**
**Correlations of personality traits with root-mean-square error**								
ρ	−0.01	0.05	0.56	−0.39	0.43	−0.19	0.00	0.52
*p*	0.96	0.84	0.02	0.13	0.09	0.48	0.99	0.04
**Correlations of personality traits with mean absolute error**								
ρ	−0.05	0.07	0.51	−0.42	0.46	−0.25	−0.01	0.49
*p*	0.83	0.80	0.04	0.10	0.07	0.35	0.97	0.06

*Presented as Spearman correlation coefficients (ρ) and *p*-values. SA, social anxiety; CE, cognitive empathy; AE, affective empathy; D, depression; diff, difference.*

**TABLE 5:** Correlations between engagement estimation errors and the means and differences in four personality traits in dyad-non-specific estimation.

	**SA mean**	**SA diff**	**AE mean**	**AE diff**	**CE mean**	**CE diff**	**D mean**	**D diff**
**Correlations of personality traits with root-mean-square error**								
ρ	0.38	0.01	0.42	0.25	0.05	0.38	0.50	0.04
*p*	0.14	0.96	0.099	0.36	0.85	0.14	0.049	0.88
**Correlations of personality traits with mean absolute error**								
ρ	0.43	0.06	0.40	0.46	−0.11	0.48	0.38	−0.08
*p*	0.096	0.83	0.12	0.07	0.68	0.056	0.14	0.76

*Presented as Spearman correlation coefficients (ρ) and *p*-values. SA, social anxiety; CE, cognitive empathy; AE, affective empathy; D, depression.*

To verify whether these correlations may be simply due to higher engagement or range of engagement in dyads with certain personality traits, Spearman correlation coefficients were also calculated between the same personality traits and median self-reported engagement within each dyad as well as the engagement range (maximum – minimum) within each dyad. A significant correlation was found between the difference in depression values and the engagement range (ρ = 0.54, *p* = 0.03). All other correlations had *p* > 0.1.

## Discussion

### Primary Analyses

In dyad-specific engagement estimation (Table 1), all machine learning methods achieved similar RMS and MA errors to the baseline error estimator. In dyad-non-specific estimation (Table 2), all machine learning methods then achieved slightly lower RMS and MA errors than the baseline estimator. Additionally, in the 12 dyads where external engagement ratings were available, the dyad-non-specific machine learning methods achieved lower errors than the external coder.

These results indicate that interpersonal engagement can be estimated from physiological responses on the level of individual dyads with some accuracy. We consider the dyad-non-specific scenario to be more realistic, as it does not assume that any data are available from the current dyad. Conversely, the dyad-specific scenario assumes that data are not only available from the current dyad, but also in the same conditions (e.g., exact same sensor placement). Thus, the dyad-non-specific results are more likely to transfer to scenarios where participants either have not extensively used the system or have not carefully calibrated it.

By demonstrating the ability to perform engagement estimation in individual dyads, our study goes beyond the state of the art, where the connection between physiological responses and interpersonal engagement has only been made on the group level (e.g., with correlation analyses) (Dikker et al., 2017; Finset and Ørnes, 2017; Bevilacqua et al., 2018; Bar-Kalifa et al., 2019; Kleinbub et al., 2019; Sun et al., 2020; Tschacher and Meier, 2020; Zheng et al., 2020). Given known data about a specific dyad, physiological responses could thus be used to, for example, dynamically track the dyad’s engagement over time during conversation or provide real-time feedback about interpersonal engagement to the dyad (see section “Potential Implementation of Real-Time Automated Engagement Feedback”), which may have benefits in applications such as education, mental health counseling, and conflict resolution.

### How Accurate Is Enough?

The main limitation of the primary analyses is that it is difficult to gauge the degree to which these results may be practically useful. The baseline error estimator makes use of known past and future engagement values, so we may argue that achieving approximately the same accuracy (as in Table 1) is already impressive. At the same time, given that the training data are labeled with known engagement values, they also make use of this information, and we could conversely argue that the physiology and machine learning are only worthwhile if they achieve a higher accuracy than the baseline estimator. In dyad-non-specific estimation, errors with machine learning methods were lower than those achieved by the baseline estimator, but only slightly (RMS error 12.2 vs. 13.7, MA error 10.4 vs. 11.4), and a follow-up paired *t*-test did not find significant differences.

Alternatively, we could argue that the estimator should be able to achieve a higher accuracy than an independent human observing the conversation. In dyad-non-specific analysis, we indeed found that the estimation algorithms were more accurate than the external coder (RMS error 11.2 vs. 15.2, MA error 9.0 vs. 11.8) and thus may be valuable. There are several caveats to this result. First, ratings were established by a single coder, introducing the possibility of systematic coding error. Second, the coder was told only to rate the dyads’ engagement according to their own opinion, and was not asked to mimic what the dyads were likely to self-report. Third, the coder did not have access to engagement data from the other dyads (while the algorithms did), and was thus not able to, e.g., estimate the expected range of reported engagement. At the same time, as the coder had access to video and audio of the conversation and had experience in interpreting human interaction, it could nonetheless be considered impressive that the algorithms were able to achieve better accuracy based on physiological data.

Even if we agree that the algorithms are more accurate than, e.g., the external observer, we must then ask whether the difference in accuracy (in that case, RMS error difference of 4.0 and MA error difference of 2.8) is large enough to be meaningful. Similar issues with contextualizing the accuracy of regression algorithms have been observed in single-user affective computing: for example, both our previous work (Novak et al., 2015) and others’ work (Rodriguez-Guerrero et al., 2013) have had difficulty determining whether obtained regression accuracies are acceptable, and this has been mentioned as a grand challenge in the state of the art of affective computing (Fairclough and Lotte, 2020). In future regression studies, a simplified approach could be to define a range of ‘acceptable’ errors (e.g., all individual errors below 10.0 are ‘acceptable’) and calculate the percentage of errors that fall outside this range, thus obtaining a regression accuracy that could be analyzed with tools such as receiver operating characteristic curves. However, this requires us to be able to define an ‘acceptable’ error, which is likely application-specific. Ultimately, engagement estimation accuracy will likely need to be evaluated by determining whether it provides actual benefits to the user, as done both in our previous single-user work (McCrea et al., 2017), our previous work on physiological synchrony in competitive gaming (Darzi and Novak, 2021), and others’ work with single-user scenarios (Rodriguez-Guerrero et al., 2013; Fairclough et al., 2015).

### Potential Implementation of Real-Time Automated Engagement Feedback

We envision one possible application of our automated engagement estimation methods that would allow us to practically estimate their usefulness: we could provide dyads with information about conversation engagement as they interact with each other, allowing them to potentially notice drops in engagement that would otherwise not be visible. Dyads could then take steps to try to increase engagement by, e.g., changing conversation topics or shifting the balance of conversation from one person to the other. Such real-time feedback has been proposed by multiple researchers, and very simple versions of it have been implemented – for example, displaying the other participants’ heart rates, respiration rates or brain waves and allowing the viewer to make their own interpretations (Frey, 2016; Liu et al., 2017; Salminen et al., 2019). By fusing information from multiple physiological signals into an overall engagement estimate, our approach may allow both more accurate and more easily interpretable feedback.

Such real-time feedback, however, would have additional technical and design challenges. For example, as all our analyses were performed ‘offline’ after data collection, we were able to manually remove artifacts such as inaccurate ECG peak detection. This would be harder in real time, and even a single artifact may lead to major errors in estimated engagement. Furthermore, engagement could be presented in different ways (e.g., graphical, numerical), which may have a major effect on how users react to it (Liu et al., 2017). Nonetheless, we believe that these challenges are solvable, and that implementing real-time feedback based on automated engagement estimation would allow researchers to better quantify acceptable accuracies and potential benefits of such engagement estimation.

### Secondary Analyses

#### Engagement Estimation Without Synchrony or Participant Characteristics

Removing synchrony features from the dataset had little effect on dyad-specific estimation, but did somewhat increase errors in dyad-non-specific estimation (RMS error 13.7 vs. 12.3, MA error 12.0 vs. 10.0). Synchrony features were also among the top three most important features for all three machine learning methods (Table 3). Removing participant characteristics also increased errors in dyad-non-specific estimation (RMS error 14.0 vs. 12.3, MA error 12.4 vs. 10.0). Since this decrease in accuracy was not large, we may ask whether it is worth including physiological synchrony metrics, which require additional computation, or personality traits, which require additional self-report questionnaires. We believe that at least participant traits are worth including since the questionnaires are not very long and the answers are likely to remain stable over time. However, discussion on this topic again runs into the limitation from the previous section that it is difficult to gauge the degree to which differences in accuracy are practically meaningful.

#### Effect of Personality on Engagement Estimation

Root-mean-square and MA errors are correlated with dyads’ personality traits in both dyad-specific and dyad-non-specific engagement estimation (Tables 4, 5), indicating that the difficulty of estimating a dyad’s engagement depends on their personality traits. In dyad-specific estimation, there are positive correlations between estimation error and cognitive/affective empathy, which appears to indicate that dyads with higher empathy are harder to ‘read.’ Additionally, in dyad-specific estimation, there is a positive correlation between estimation error and the difference between participants’ depression values, indicating that dyads are harder to ‘read’ if there is a discrepancy in depression between members of the dyad. In dyad-non-specific estimation, there is a positive correlation between error and depression; this would indicate that depressed dyads are harder to ‘read.’

The correlation with depression may simply be because dyads with a bigger difference in depression values have a higher range of engagement, increasing the possible error; however, this was not observed for the other personality traits. Thus, if these observations are accurate, they may have important implications for practical usage of automated engagement estimation from physiology, as they would indicate that such estimation is likely to be more accurate for certain types of dyads. However, given the small sample size and secondary nature of the analysis, these results may also be due to statistical noise.

### Alternative Study Protocols

The current study protocol was one possible approach to automatic engagement estimation in conversation. In this section, we describe alternative possibilities that could involve either modifications to the current approach (see section “Modified Single-Session Regression Scenario”) or an entirely different approach (see section “Classification and Multi-Day Scenarios”).

#### Modified Single-Session Regression Scenario

Based on experience from the current study, we can suggest some modifications to improve the quality of the 15-min uninterrupted conversation scenario. First, we took the mean of the two participants’ engagement value to obtain dyad engagement. This does not account for the possibility that one participant may consider the conversation to be much more or less engaging than the other participant – for example, individual ratings of 0 and 100 would not be distinguishable from individual ratings of 50 and 50. Such large disagreements occurred in two of our dyads, which we then removed from the dataset (see section “Participants”); however, in the future, we may consider determining different scenarios based on individually reported engagement rather than simply averaging.

Second, the currently used engagement scale was a single visual analog scale ranging from “none” to “very high,” with the markings converted to 0–100 values. It is unlikely that participants can distinguish variation in engagement on a very granular level – e.g., between 90 and 95. We may instead consider, e.g., a scale with numbers between 1 and 10, with anchors at 1, 3, 5, 7, and 10 to improve reliability and consistency. We could also consider a multi-item scale, such as the IIQ (Goršič et al., 2019) or the Flow Short Scale used in another recent automatic engagement estimation study (Carroll et al., 2020), but this would make it difficult to maintain free-flowing conversation.

Third, we could choose to omit self-reported engagement entirely and focus only on externally rated engagement, as suggested by some non-dyadic psychophysiology studies (Schwerdtfeger, 2004). In this case, we would recruit two or three coders and train them more extensively in engagement rating until they have reached a certain consistency, as done in our previous study on IIQ questionnaire validation (Goršič et al., 2019) and in other studies of physiological linkage (McKillop and Connell, 2018). In this case, we would obtain more consistent engagement ratings, though we may not be able to identify any internal processes that may affect physiological responses but are not externally visible to the coders.

Fourth, physiological processes do not necessarily instantly react to changes in conversation engagement. Thus, we could consider estimating engagement not only based on physiological features from the same 60-s interval, but also based on physiological features from an adjacent interval, as suggested by other researchers (Muszynski et al., 2018). While this may violate causality (by estimating current engagement from future measurements) and not be suitable for real-time feedback, it may have additional fundamental insights.

Finally, to potentially enhance dyad-non-specific estimation, we could consider different or additional baseline intervals. For example, some dyadic studies have had participants face their partner with eyes open rather than closed (Pan et al., 2020), and some have used multiple baselines where participants first face a wall and then each other (Bevilacqua et al., 2018).

#### Classification and Multi-Day Scenarios

In the current study, we used a protocol with uninterrupted, free-flowing conversation, as this would more closely approximate real-world scenarios. We acknowledge that this resulted in a limited range of engagement within each dyad (see section “Summary of Conversations”), making it relatively easy to obtain a high accuracy with the baseline estimator (taking the median of training engagement values). We nonetheless believe that our study is valuable and that the proposed methods would generalize to scenarios with a broader range of engagement. However, in the future, we will also explore an alternative study protocol where dyads will go through multiple artificially induced conversation scenarios (e.g., told to argue with each other), and classification methods will be used to assign physiological data to one of the possible scenarios. While less natural than the current protocol, this is likely to provide more balanced data, and classification algorithms are more common than regression algorithms in both studies of physiological synchrony (Hernandez et al., 2014; Konvalinka et al., 2014; Muszynski et al., 2018; Zhu et al., 2018; Brouwer et al., 2019; Verdiere et al., 2019; Pan et al., 2020; Darzi and Novak, 2021) and general affective computing (Novak et al., 2012; Aranha et al., 2019).

Unrelated to the above classification approach, we may also consider a multi-day protocol where engagement estimation algorithms are trained on data from one session, then tested on data from another session. While more time-consuming, this would demonstrate the stability of physiology-based engagement estimation over time. For example, if the estimation error significantly increases when testing on a different session (as opposed to the same session), this would indicate that the estimation algorithms need to be recalibrated in each session, significantly decreasing their usefulness.

### Additional Measurements

In the current study, we used a set of physiological measurements that are relatively easy to self-apply. This decision was taken due to the COVID-19 pandemic, which precluded the use of more complicated measurements such as electroencephalography. We believe that the focus on these measurements is realistic, as they are relatively simple and thus more likely to be used in an applied context; furthermore, they are common in group-level analyses of physiological synchrony (Hernandez et al., 2014; Finset and Ørnes, 2017; McKillop and Connell, 2018; Muszynski et al., 2018; Bar-Kalifa et al., 2019; Steiger et al., 2019; Tschacher and Meier, 2020). Still, in the future, we may explore two additional physiological measurements: electroencephalography, which is common in studies of physiological synchrony (called hyperscanning in the domain of brain measurements) (Konvalinka et al., 2014; Dikker et al., 2017; Pérez et al., 2017; Bevilacqua et al., 2018; Ding et al., 2021), and functional near infrared spectroscopy, which is similarly common in synchrony studies (Sun et al., 2020; Zheng et al., 2020). We may also explore the addition of non-physiological measurements, such as gesture synchronization and analysis of voice patterns.

## Conclusion

This paper presents the use of machine learning algorithms combined with physiological measurements to estimate interpersonal engagement during a 15-min conversation. These machine learning algorithms were able to estimate self-reported engagement with an accuracy similar to a baseline estimator in dyad-specific estimation, and with an accuracy slightly higher than the baseline estimator in dyad-non-specific estimation. Additionally, in dyad-non-specific estimation, they achieved a higher accuracy than a human observer. However, these results are difficult to contextualize, as it is unclear whether such an accuracy would be practically acceptable, and would need to be evaluated for usefulness in a more practical setting.

In secondary analyses, we showed the effects of removing physiological synchrony features and participant characteristics from the dataset. Additionally, we showed that the engagement estimation error is correlated with personality traits, indicating that some types of dyads are harder to ‘read.’

Overall, this paper presents the feasibility of dynamically estimating a dyad’s interpersonal engagement during a naturalistic conversation. Similar approaches could be used as a complement to self-report measures and external observation when analyzing communication scenarios. They could also be used as a basis for real-time feedback: providing dyads with information about interpersonal engagement, allowing them to take steps to increase it. However, further work is needed to identify acceptable accuracies in such situations.

## Data Availability Statement

The raw data supporting the conclusions of this article will be made available by the authors, without undue reservation.

## Ethics Statement

The studies involving human participants were reviewed and approved by the University of Wyoming Institutional Review Board. The patients/participants provided their written informed consent to participate in this study.

## Author Contributions

IC contributed to the study design, performed the all physiological data analysis, and contributed to manuscript writing. MG contributed to data collection and non-physiological data analysis. JC contributed to the literature review, study design, and manuscript writing. DN supervised the entire study, led the study design and literature review, contributed to data collection, and wrote the majority of the manuscript. All the authors read and approved the final manuscript.

## Conflict of Interest

The authors declare that the research was conducted in the absence of any commercial or financial relationships that could be construed as a potential conflict of interest.

## Publisher’s Note

All claims expressed in this article are solely those of the authors and do not necessarily represent those of their affiliated organizations, or those of the publisher, the editors and the reviewers. Any product that may be evaluated in this article, or claim that may be made by its manufacturer, is not guaranteed or endorsed by the publisher.
